# Plasma and feces multiomics unveil cognition‐associated perturbations of chronic inflammatory pathways of the gut‐microbiota–brain axis

**DOI:** 10.1002/alz.70844

**Published:** 2025-10-30

**Authors:** Farideh Hosseinkhani, Claire Chevalier, Moira Marizzoni, Rahel Park, Sabine Bos, Alida Kindt Dunjko, Cornelia M. van Duijn, Amy C. Harms, Giovanni B. Frisoni, Thomas Hankemeier

**Affiliations:** ^1^ Leiden Academic Centre for Drug Research Leiden University Leiden Netherlands; ^2^ Laboratory of Neuroimaging of Aging (LANVIE) University of Geneva Geneva Switzerland; ^3^ Geneva Memory Center, Department of Rehabilitation and Geriatrics Geneva University Hospitals Geneva Switzerland; ^4^ Laboratory of Biological Psychiatry IRCCS Istituto Centro San Giovanni Di Dio Fatebenefratelli Brescia Italy; ^5^ Department of Epidemiology Erasmus University Medical Center Rotterdam Netherlands

**Keywords:** Alzheimer's disease, gut microbiota, gut‐brain axis, metabolomics, metagenomics

## Abstract

**INTRODUCTION:**

Gut‐microbiota dysbiosis has been linked to cognitive decline. Given its role in metabolism, immunity, and environmental interactions, broader molecular signaling alterations are likely.

**METHODS:**

We analyzed gut microbiota composition, plasma and fecal metabolites, and inflammatory cytokines across cognitive stages, from healthy controls to dementia.

**RESULTS:**

Alpha diversity declined with increasing cognitive impairment severity. Short‐chain fatty acid–producing Firmicutes and Bacteroidota decreased from 76% and 17% in controls to 59% and 11% in dementia, respectively. Proteobacteria (e.g., Escherichia‐Shigella) rose from < 2% to 4%, and Verrucomicrobiota from 3% to 11%. Despite overall Firmicutes decline, *Ruminococcus gnavus*, a mucus‐degrading species, increased in dementia. These shifts correlated with elevated plasma cytokines, suggesting a link between gut dysbiosis and systemic inflammation. Bacteria‐associated metabolites, including bile acids, trimethylamine N‐oxide, oxylipins, sugars, and fatty acids were significantly altered. Changes were seen as early as subjective cognitive decline.

**DISCUSSION:**

Larger studies are needed to validate these findings and explore microbiome‐based interventions.

**Highlights:**

- Examined gut microbiota, inflammation, and metabolic changes in cognitive impairment stages
- Early metabolic changes in feces detected before plasma alterations
- Observed shifts in gut microbiota and inflammation associated with cognitive decline
- Suggests potential for early biomarkers based on gut metabolites
- Calls for larger, longitudinal studies to validate findings

## 1 INTRODUCTION

Cognitive impairment (CI) and dementia affecting millions worldwide, pose significant challenges for diagnosis, prognosis, and prevention.^1^ Current research recognizes diverse pathophysiological factors, including inflammation, neurological damage, and metabolic changes, all influencing CI development.^2^ The gut microbiota, comprising trillions of microbes in the human gastrointestinal tract, has emerged as a significant dimension in this complex neurological condition.

Extensive evidence links gut microbiota dysbiosis to CI.^3^ Analyses of 16S ribosomal RNA (16S rRNA) sequences consistently reveal an altered Bacteroidetes‐to‐Firmicutes ratios and reduced overall microbial diversity in CI patients.^4^, ^5^ Additionally, correlations exist between apolipoproteint E (ApoE) genotypes, a known CI risk factor, and specific gut bacterial composition, including Prevotellaceae and Ruminococcaceae families.^6^ Furthermore, mouse models show microbiota modulation can mitigate neurodegeneration, often ApoE isoform dependent, highlight the possible intricate link between genetics, gut microbiota, and CI pathogenesis.^7^

In the past decade, analytical advances have unveiled the bidirectional molecular communication between gut microbiota and host metabolism, impacting CI pathology. Marizzoni et al.^8^ demonstrated links between amyloid pathology, plasma pro‐inflammatory cytokines, and bacterial‐derived short‐chain fatty acids (SCFAs) in individuals with CI. Changes in bile acids composition, including secondary bile acids produced through microbial enzymatic activity in the gut, have been reported.^9^, ^10^ Baloni et al. supported these findings, proposing the intriguing transport of secondary bile acids from the gut to the brain, as their presence cannot be solely explained by enzymatic activity within the brain.^11^ Additionally, microbial‐derived neurotransmitters like serotonin, dopamine, and gamma‐aminobutyric acid (GABA) have been implicated in CI, influencing inflammatory signaling and cognitive function.^12^

The microbiota's responsiveness to environmental factors, such as diet, suggests its influence extends across multiple signaling pathways. Unravelling these interactions could clarify mechanisms and guide gut‐brain axis therapy. While metabolic dysregulation in dementia and mild cognitive impairment (MCI) has been extensively studied, less attention has been given to subjective cognitive decline (SCD), a mixed population with cognitive complaints not yet objectified by neuropsychological tests, but at risk for developing CI.^13^ Early microbial and metabolic signatures in SCD could reveal preventive targets.

In this study, we aimed to gather immune status, microbiome composition, metabolome data, and clinical metadata from individuals across cognitive stages. We analyzed metabolites from both feces and plasma in the same individuals to gain insight into systemic molecular changes and examined how these changes correlate with the gut microbiota profile. Additionally, by including individuals with SCD, we investigated early alterations in metabolism and gut microbiota that might contribute to the onset of CI. The physiological mechanisms linking the gut microbiota to the observed metabolic alterations should be further investigated in larger cohorts to elucidate their potential therapeutic implications.

## 2 MATERIALS AND METHODS

### 2.1 Sample information

Participants in this study come from the Geneva Memory Center, representing a range of cognitive states, from unimpaired to dementia. This research project includes healthy volunteers and Memory clinic patients with cognitive complaints. Patients received diagnostic assessments, including clinical, neuropsychological evaluations, and relevant biomarker investigations.^14^ Participants were categorized into different cognitive stages: cognitively unimpaired (referred to as healthy control [HC] in this study), SCD^15^, MCI^16^, and dementia.^17^ The criteria for SCD, MCI, and dementia stages were based on their respective clinical diagnostic criteria. Sociodemographic data, including age and sex, were collected for all participants. Global cognition was assessed using the Mini‐Mental State Examination (MMSE). Additionally, participants provided blood and fecal samples, totaling 278 plasma samples and 267 fecal samples for this analysis (Table 1). Written informed consent was obtained from all participants, and the study received approval from the Geneva Ethics Committee (CCER_2016‐01346 and CCER_2020_00403).

**TABLE 1 alz70844-tbl-0001:** Demographic characteristics of participants

Parameter	HC	SCD	MCI	Dementia
Age	63 ± 10	69 ± 7	71 ± 8	69 ± 8
Gender (F/M)	57/29	37/ 26	51/60	8/9
MMSE score	29 ± 2	28 ± 2	26 ± 2	21 ± 4
Plasma (no.)	87	63	111	17
Feces (no.)	83	63	107	14

*Note*: Data have been presented in Mean ± standard deviation unless otherwise mentioned.

Abbreviations: HC, healthy control; MCI, mild cognitive impairment; MMSE, Mini‐Mental State Examination; SCD, subjective cognitive decline.

### 2.2 Metagenomics

DNA was extracted from 180 to 200 mg of frozen feces with a MagPurix Bacterial DNA extraction kit (cat ZP 02006, Zinexts) according to the manufacturer instructions. Prior to DNA extraction, fecal samples were mechanically disrupted[Table alz70844-tbl-0001] with bead‐beating using Precellys lysing kit soil grinding SK38, 3 × 40 s at 6000 rpm, followed by 5 min at 95°C shaking at 600 rpm. DNA was quantified using a NanoDrop ND‐1000 spectrophotometer, and then stored at −20°C until subsequent analyses. The regions V3 and V4^40^ of the bacterial 16S rRNA gene was amplified and purified according to 16S Metagenomic Sequencing Library Preparation protocol by Illumina. The sequencing was performed on an Illumina Miseq Sequencer with 300 bp paired‐end run using MiSeq V3 reagent with 10% PhiX and loading concentration of 6pM. Raw sequencing data from the bacterial assay (fastq format) was demultiplexed and processed into amplicon sequence variants (ASVs) with qiime2 (version 2021.8.0)^18^ using dada2 (v1.24.0)^19^ for reads quality filtering and merging. Silva138.1 SSU Ref NR99 database was formatted with QIIME2 rescript plugin and adapted to 16S V3‐V4 region and thereafter used to classify the reads with feature‐classifier plugin from QIIME2 (classifysklearn method).^20^ Sequences classified as mitochondria, chloroplasts or eukaryota, and ASVs without phylum level assignment were filtered out from the data sets.

### 2.3 Immunoassay

To quantify plasma circulating cytokine levels, we employed the analytically validated Meso Scale Discovery (MSD) V‐PLEX Human Proinflammatory Panel II (4‐PLEX) kit (IL‐1β, IL‐6, IL‐8, TNF‐α, #K15053D‐1), following the manufacturer's protocol. All plasma samples were measured after a two‐fold dilution in a single assay. Assay reproducibility was evaluated through analysis of standard replicates and pooled quality controls (QCs) plasma samples were run across four measured batches to better capture biological variability. The system exhibited excellent well‐to‐well reproducibility and sensitivity, as evidenced by standard deviation of replicates (%CVs) below 10% and the lower and upper limits of detection (Table S1 and Figure S1). To analyze the multiplex data, we utilized the MSD Discovery Workbench analysis software.

**RESEARCH IN CONTEXT**

1. **Systematic review**: A thorough review of existing literature was conducted to understand the role of gut microbiota, inflammatory cytokines, and metabolic pathways in cognitive impairment. While previous studies have focused on individual factors, there is limited research examining the interplay between these elements at various stages of cognitive decline especially early stage of subjective cognitive dementia (SCD).
2. **Interpretation**: Our findings indicate that changes in gut microbiota, inflammatory markers, and metabolic pathways occur as cognitive decline progresses and detectable in SCD stage. In particular, we observed early alterations in fecal metabolites compared to plasma, which may suggest the potential of gut‐related markers in early diagnosis or disease monitoring.
3. **Future directions**: Future studies should focus on validating these findings with larger cohorts and over longer timeframes. Longitudinal studies could further explore whether gut microbiota and metabolic changes could serve as early biomarkers for cognitive decline and Alzheimer's disease.

### 2.4 Metabolomics

#### 2.4.1 Chemicals and reagents

Analytical grade methanol (MeOH) was purchased from Biosolve (Biosolve BV, Valkenswaard, The Netherlands). Ethyl acetate, methyl tert‐butyl ether (MTBE), butanol, ammonium formate (≥99.995%), ammonium hydroxide (28−30 wt % solution of ammonia in water) sodium chloride (≥99.0%), and sodium hydroxide were obtained from Sigma–Aldrich (Sigma–Aldrich, Burlington, WV, USA). MilliQ water was sourced from a Merck Milli‐pore A10 purification system (Raleigh, NC, USA). Information regarding the stable isotope‐labeled standards and their respective supplier has been provided in our previous publications.^22^, ^23^

#### 2.4.2 Sample extraction

Fecal matter and plasma samples were aliquoted into smaller volumes and subjected to extraction for metabolome analyses. Liquid‐liquid extraction, as previously described by our research group^23^ with minor modifications for plasma samples, was employed for processing polar metabolites. In‐house standard operational procedures were followed for the extraction of lipids. Additional information regarding the extraction methods for each specific biospecimen can be found in the Supplementary File.

#### 2.4.3 Analytical measurements

To perform the chromatographic separation of polar metabolites, we utilized the SeQuant ZIC‐c HILIC HPLC column (2.1 mm × 100 mm, 3.0 µm, Merck, Darmstadt, Germany) on a Waters Acquity Ultra high‐performance liquid chromatograph (LC) (Duisburg, Germany). The chromatography method was adapted from a previously described procedure.^22^ Mobile phase A consisted of 90% acetonitrile and 10% water with 5 mM ammonium formate, while mobile phase B comprised 10% acetonitrile and 90% water with 5 mM ammonium formate. An injection volume of 3 µL was used, with a flow rate of 0.5 mL/min. The gradient for the separation was as follows: 0 min−0% B, 2 min−15% B, 5 min−21% B, 7.5 min−26% B, from 10 to 11 min−40% B, followed by re‐equilibration at 0% B from 11.5 to 18 min to restore column conditions. Mass spectrometry experiments were performed using a quadrupole‐time of flight (TOF) instrument (SCIEX 6600+ TripleTOF, AB SCIEX, Foster City, CA). Electrospray ionization (ESI) was utilized in both positive and negative ion modes, with the following ESI source parameters (positive/negative ion mode): spray voltage ± 4.5 kV, capillary temperature 400°C, sheath gas 20, auxiliary gas 25, and curtain gas 25. Full scan mode was employed for data acquisition across a m/z range of 50–900 Da.

For profiling signaling lipids, an in‐house validated analytical assay was employed with minor adjustments to the flow rate (0.1 mL/min). The LC‐MS/MS protocol allowed for the relative quantitation of oxylipins derived from the conversion of omega‐6 polyunsaturated fatty acids (PUFAs), including linoleic acid (LA), dihomo‐γ‐linolenic acid (DGLA), and arachidonic acid (AA), as well as omega‐3 PUFAs such as α‐linolenic acid (ALA), eicosapentaenoic acid (EPA), and docosahexaenoic acid (DHA).^21^

To ensure data quality, the in‐house developed mzQuality workflow was utilized to correct for inter‐batch variations, with QC and blank samples were injected alongside the study samples in all measurements. Metabolites exhibiting a relative standard deviation of no more than 30% in the corrected peak area of QC samples were selected for export and subsequent analysis.

## 3 DATA PROCESSING, STATISTICS, AND VISUALIZATION

All statistical data analysis and plots were performed using R (version 4.2.1). For sequencing data, the phyloseq (v1.40.0) were used.^24^ A prevalence threshold of 10% was implemented, retaining only those ASVs that are present in a minimum of 10% of the overall samples. Alpha diversity was estimated on ASV level using Shannon's and Chao1 index, and significance (*p*‐value < 0.05) was assessed using non‐parametric Wilcoxon tests. Shannon index represents microbial diversity within samples, while Chao1 index estimates species richness. To estimate beta diversity, abundances (counts) were centered log‐ratio transformed (clr), and distances were calculated. Principal Coordinates Analysis (PCoA) was performed using the Weighted UniFrac metric, which considers both taxonomic composition and relative abundance of ASVs. To identify taxa with differential abundance between each cognitive decline status and HC, the Linear Discriminant Analysis Effect Size (LEfSe) algorithm was employed (*p*‐value <  0.05 and LDA  >  2). LEfSe combines Kruskal–Wallis and Wilcoxon rank‐sum tests to detect significant differences in relative abundance, followed by linear discriminant analysis to estimate effect size of each differentially abundant taxon.

Metabolomics data were imputed using predictive mean matching from the “mice” package (version 3.15.0), accessible at https://rdocumentation.org/packages/mice/versions/3.15.0. The parameters for number of imputations and iterations were 20 and 15, respectively. Fisher's exact test was used to evaluate the significance of missing values across various cognitive statuses before imputation. Additionally, samples with over 50% missing metabolite data were excluded from further analysis (Table S2). Furthermore, the variables underwent normalization, log (log 2 scale)‐transformation, with normality assessed through Normal Q‐Q and Residuals versus Fitted value plots.

Group comparisons were performed using analysis of variance (ANOVA) with Bonferroni correction for continuous Gaussian variables, Kruskal–Wallis test followed by Dunn's correction for non‐Gaussian variables, and chi‐squared test for categorical data. Significant differences between each CI status and HC were further evaluated using linear regression models adjusted for age and gender as covariates. Differential metabolite expression between each CI group and HC was visualized using volcano plots. Metabolites with an absolute estimate greater than 1.25 and a *p*‐value below 0.05 were selected for further analysis. To control the false discovery rate (FDR) in these multiple metabolite comparisons, the Benjamini–Hochberg procedure was applied with an FDR threshold of 0.15.

To assess the correlation between plasma and feces metabolites, Spearman correlation coefficients were calculated. Additionally, linear regression models were utilized to evaluate the correlation between immune cytokines and the metadata listed in Table 1. The correlation analysis between metabolites, immune cytokines, and differentiative bacteria (average relative abundance more than 0.1%) was conducted using the Spearman correlation coefficient.

## 4 RESULTS

In total, 278 plasma and 267 feces samples were included in this study. The clinical characteristics of the study population are presented in Table 1.

### 4.1 Plasma inflammatory cytokines

**FIGURE 1:**
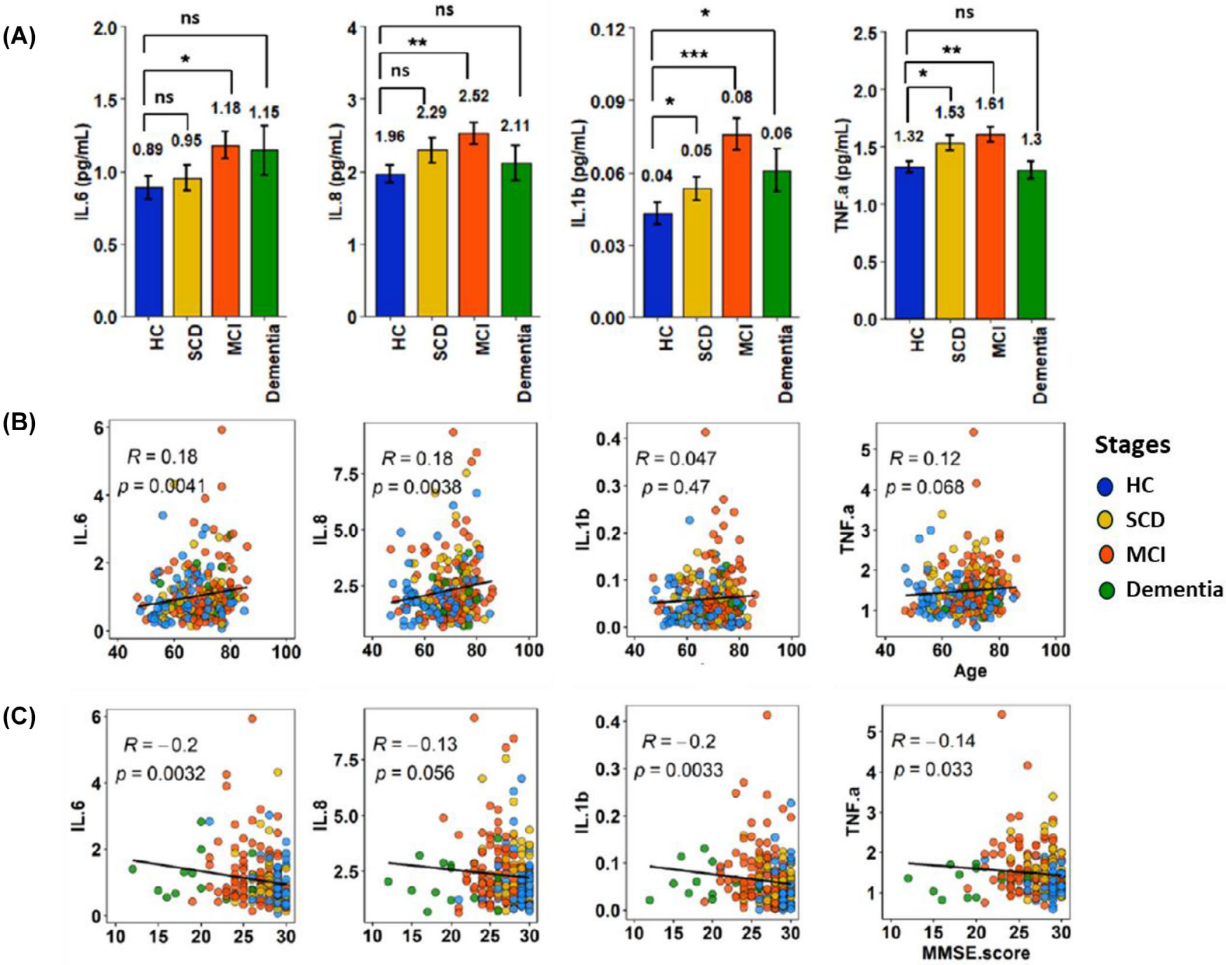
(A) Plasma proinflammatory cytokine levels in different states of cognitive impairment in comparison to healthy control (B) Linear regression analysis of age and circulating cytokines concentrations; (C) Linear regression analysis of clinical MMSE score and circulation cytokines concentrations. q‐Values were calculated by using Kruskal–Wallis test followed by Dunn's post hoc correction for multiple comparisons. HC, healthy control; IL, interleukin; MCI, mild cognitive impairment; MMSE, Mini‐Mental State Examination; SCD, subjective cognitive decline; TNF.a: tumor necrosis factor‐alpha. *** *q*‐value < 0.001, ** *q*‐value < 0.01, * *q*‐value < 0.05

We assessed the inflammatory status by measuring four pro‐inflammatory cytokines: Tumor necrosis factor alpha (TNF‐α), interleukin (IL) ‐6, IL‐8, and IL‐1β (Figure 1A and Tables S1 and S3). Cytokine levels increased progressively from the SCD stage, though not significantly compared to the HC. In the MCI group, all four cytokines were significantly higher (*p* < 0.05) than in HC. In dementia, only IL‐1β showed a marginal significant increase (*p* = 0.08). Linear regression analyses revealed no significant correlations between cytokines levels, age, or MMSE scores (Figure 1B; Figure S2). Effect sizes were modest, potentially limited by sample size in advanced stages.

### 4.2 Gut microbiota community

**FIGURE 2:**
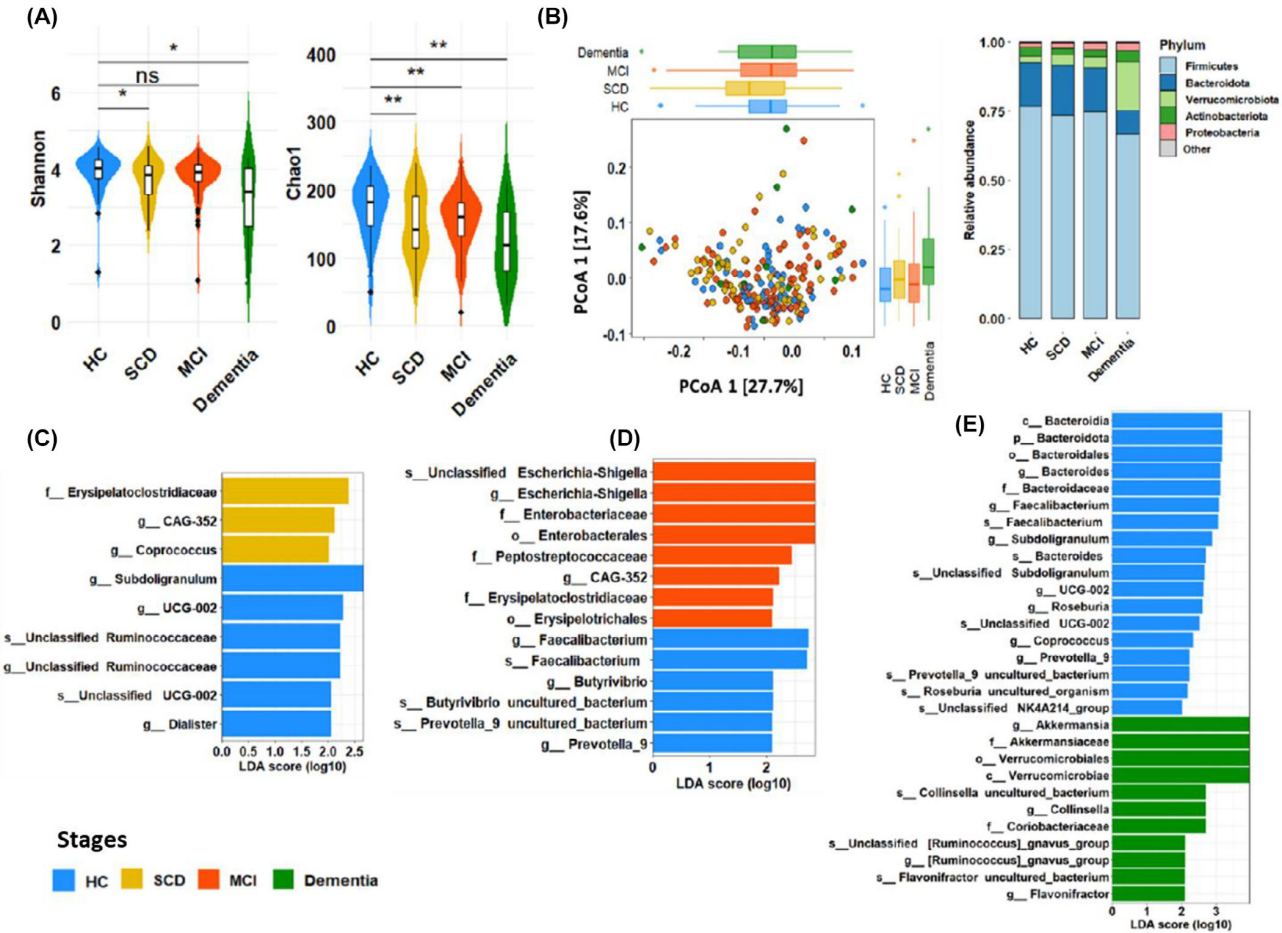
Microbiome compositional analysis of individuals in different status of cognitive impairment. (A) The Shannon and Chao1 index representing significant decrease of species richness between different states of cognitive impairment in comparison to healthy control. (B) Beta diversity based on weighted UniFrac visualized using Principal Coordinates Analysis (PCoA) which shows differences in microbial community composition across samples. (C) Linear Discriminant Analysis (LDA) Effect Size (LEfSe) between HC (blue) and SCD (yellow), where the LDA score indicates the magnitude of group differences in bacterial taxa.), (D) LEfSe between HC and MCI (red), (E) LEfSe between HC and dementia (green). HC, healthy control; MCI, mild cognitive impairment; SCD, subjective cognitive decline. *** *p*‐value < 0.001, ** *p*‐value < 0.01, * *p*‐value < 0.05

16S rRNA analysis revealed varying total bacterial taxa abundances across CI stages (Supplementary Table S4). Alpha diversity metrics (Shannon and Chao1), declined significantly in CI groups versus HC, indicating reduced gut microbiota richness (Figure 2A). Beta diversity analysis via PCoA with the Weighted Unifrac metric showed no significant compositional differences between groups (Figure 2B). The distribution of the five dominant phyla showed a progressive decline in Firmicutes from 76% in HC to 59% in dementia, while Actinobacteria (2% to 15%), Verrucomicrobiota (3% to 11%), and Proteobacteria (2% to 4%) increased, starting from the SCD stage (Figure 2C, Figure S3).

The LEfSe analysis identified taxa with differential abundance (Figure 2D–F). HC exhibited higher abundance of SCFA‐producing genera in Firmicutes (UCG‐002, *Faecalibacterium*, Ruminococcaceae, *Butyrivibrio*, *Subdoligranulum*) and Prevotella_9 (Bacteroidota). Conversely, opportunistic pathogens such as *Escherichia‐Shigella* and *R. gnavus* increased with CI severity.

### 4.3 Metabolomics

**FIGURE 3:**
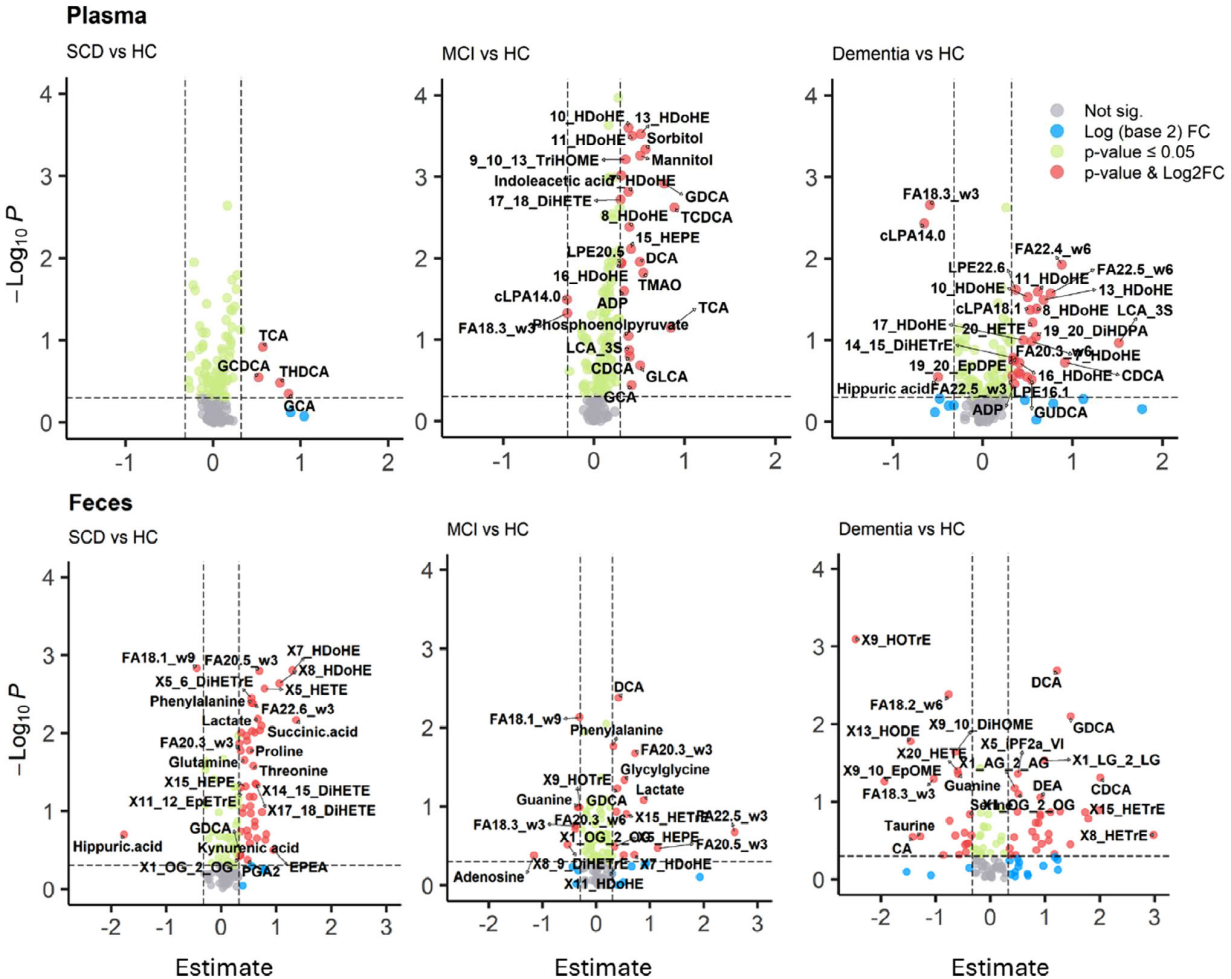
Volcano plots utilizing linear model estimate to compare metabolites identified in both plasma and fecal matrices of individuals across different cognitive impairment statuses against the HC group. Metabolites meeting the criteria of having *p*‐values below 0.05 and an absolute Estimate greater than 1.25‐fold are highlighted and labeled in red. Notably, metabolites increased in the respective cognitive impairment statuses are represented by red dots on the right side of each plot, while metabolites that decreased compared to HC are represented by red dots on the left side. HC, healthy control; MCI, mild cognitive impairment; SCD, subjective cognitive decline

Targeted metabolomic profiling of plasma and feces identified 183 metabolites in plasma and 141 in feces, spanning various chemical classes including bile acids, fatty acids, oxylipins, endocannabinoids, amino acids, sugars, organic acids, etc. Metabolites alterations are illustrated in Figure 3 (see Tables S5 and S6). Significant alterations were observed in metabolites associated with bacterial enzymatic activities, including bile acids, trimethylamine N‐oxide (TMAO, plasma only), and tryptophan, with changes evident from the SCD stage (*p* < 0.05). As CI progressed, pronounced alterations occurred in fatty acids, oxylipins, sugars, and amino acids, with similarities between plasma and fecal samples. Notably, significant alterations in conjugated bile acids, fatty acids, and oxylipins (e.g., x‐DiHETEs, x‐HDOHEs) were observed in fecal samples during the SCD stage, preceding corresponding changes in plasma.

Spearman correlation analysis revealed positive correlations between fecal and plasma metabolite levels, particularly for oxylipins, secondary bile acids, long‐chain fatty acids, and glucose (Figure S4). Specific long‐chain fatty acids (e.g., 22:4n‐6, 22:5n‐6 in plasma; 20:3n‐3, 20:5n‐3, 22:5n‐3, 22:6n‐3 in feces) increased, while dietary fatty acids (linoleic acid, α‐linolenic acid, oleic acid) decreased with CI progression. Elevated levels of lipoxygenase (LOX)‐derived oxylipins such as HDOHEs, HETEs, HEPEs were consistent in both matrices. Endocannabinoids, including acyl‐glycerols (2‐AGs, 2‐OG, 2‐LG), showed significant changes only in fecal samples.

### 4.4 Association of microbial genera with inflammatory cytokines and metabolic alteration in plasma and feces

**FIGURE 4:**
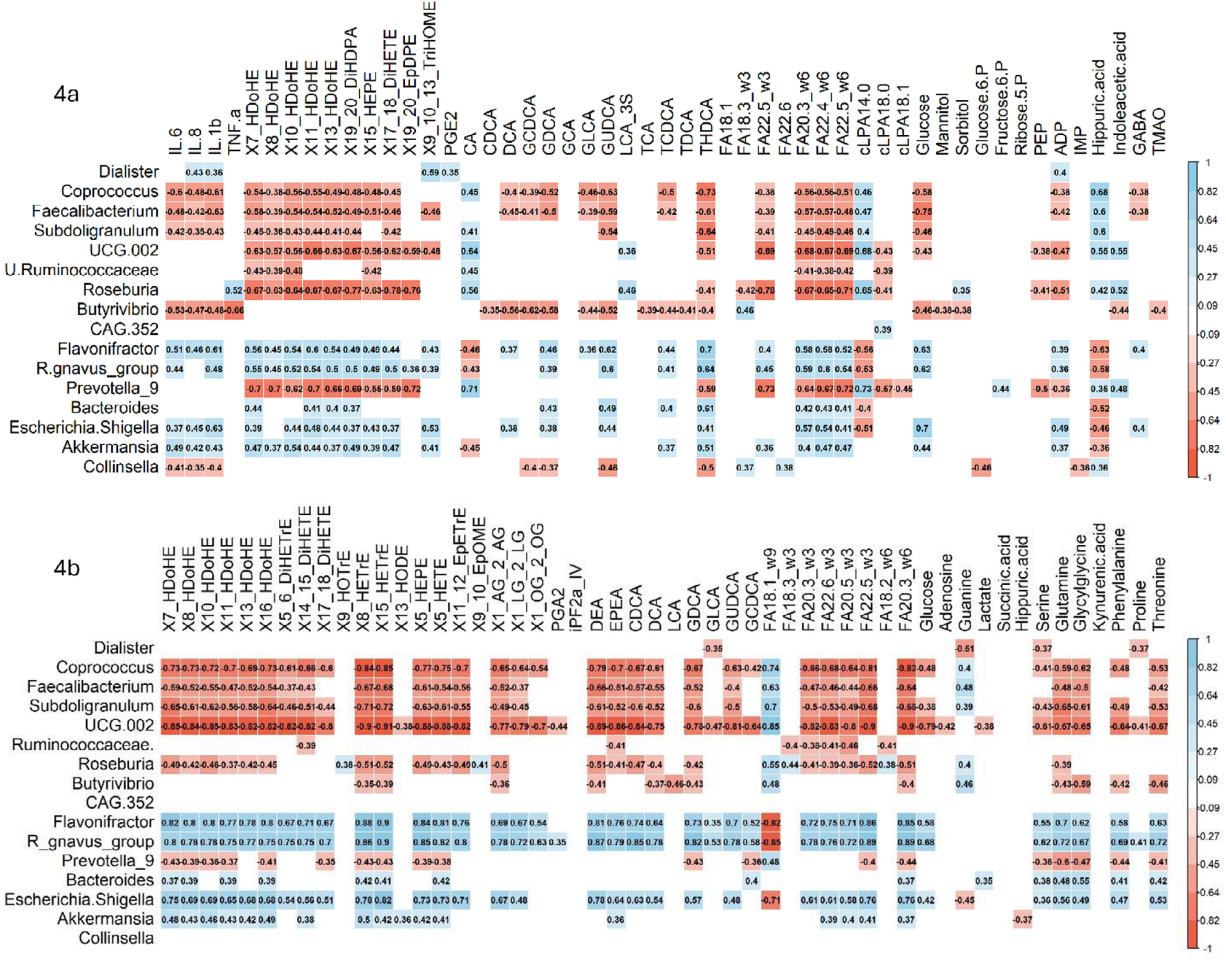
Spearman correlation analyses investigating associations between alterations in plasma (4A) and fecal metabolites (4B), along with plasma cytokine levels, and the abundance of bacteria exhibiting significant changes within each cognitive impairment status

Spearman correlation analyses explored associations between plasma and fecal metabolites, plasma cytokine levels, and bacterial genera with significant changes across CI statuses (Figure 4A–B, Tables S7 and S8). Elevated plasma cytokines were associated with reduced SCFA‐producing bacteria (*Faecalibacterium*, *Coprococcus*, *Bacteroides*, *Roseburia*, *Ruminococcus*) and increased opportunistic bacteria (*Escherichia‐Shigella*, *R*. *gnavus)*. These microbial shifts correlated significantly with metabolic changes in oxylipins, long‐chain fatty acids, and glucose in both plasma and fecal samples.

## 5 DISCUSSION

This study illuminates the complex relationships among gut microbiota dysbiosis, systemic inflammation, and metabolic alterations across cognitive CI stages, from SCD to dementia. Our observed reduction in gut microbiota richness, evidenced by decreased alpha diversity, aligns with prior research linking loss of microbial diversity to neurodegenerative disorders.^25^ We found a progressive shift in gut microbial composition, characterized by reduced Firmicutes and increased Actinobacteria, Verrucomicrobiota, and Proteobacteria. Such patterns, particularly the expansion of Proteobacteria and Verrucomicrobiota (e.g., *Akkermansia*) alongside the loss of SCFA‐producing Firmicutes genera are consistently associated with a pro‐inflammatory gut environment in dementia and related models. In our cohort, CI groups showed enrichment of opportunistic pathogens (*Escherichia–Shigella*, *R. gnavus*), further supporting a shift toward a pro‐inflammatory microbial profile.^27^, ^28^ Although *R. gnavus* is a natural member of the Firmicutes phylum, it can bloom under inflammatory conditions, highlighting the importance of considering strain‐level differences when assessing microbial contributions to disease.^28^

Our previous work on this sample set demonstrated reduced fecal concentrations of SCFAs such as butyrate and propionate in CI compared with HCs. This functional loss complements the observed decline in SCFA‐producing taxa and reinforces the proposed role of SCFAs in maintaining gut barrier integrity, modulating neuroinflammation, and supporting gut–brain axis communication.^8^, ^30^

We observed significant elevations of pro‐inflammatory cytokines in the MCI group, whereas changes in dementia were less consistent. This pattern may be influenced by the smaller dementia sample size, but it may also reflect stage‐specific immune dynamics. While some studies^31^, ^32^, ^33^ have reported inverse associations between cytokine levels and cognitive performance, our cohort showed no such relationship, underscoring the need for larger, longitudinal cohorts with harmonized immunophenotyping to determine whether peripheral cytokines predict progression or reflect disease stage.

Metabolomic profiling revealed distinct patterns of change across biofluids and cognitive stages. Alterations in bile acids were evident in both plasma and feces from the SCD stage, whereas plasma alteration in other metabolites appeared later, from MCI onward. Other fecal metabolites, including fatty acids, amino acids, tryptophan derivatives, and organic acids, were altered from SCD, suggesting that shifts in the gut lumen metabolome occur early in the CI continuum. The earlier and more pronounced changes in feces compared to plasma, along with the positive associations between plasma and fecal metabolites, support the idea that the intestinal environment may be a primary site of metabolic perturbation. Prior studies have linked altered bile acid metabolism to CI and proposed microbiome–bile acid interactions as potential contributors to brain pathology, although the mechanistic pathways remain incompletely understood.^9^, ^34^, ^35^

A central mechanistic thread emerging from our data is the altered fatty acid landscape and its downstream conversion into oxylipins, bioactive lipid mediators with immunomodulatory and vascular effects. Dietary and structural fatty acids (linoleic acid, α‐linolenic acid, oleic acid) decreased with CI progression, while several longer‐chain fatty acids (e.g., 22:4n‐6, 22:5n‐6 in plasma; 20:3n‐3, 22:6n‐3 in feces) increased. One possible interpretation is that neuronal and membrane injury releases long‐chain PUFAs from phospholipids into circulation and the gut lumen, where they become substrates for COX‐, LOX‐, and CYP‐mediated oxygenation to oxylipins. In line with this, LOX‐derived oxylipins (HDOHEs, HETEs, HEPEs) were elevated in both plasma and feces, consistent with previous work linking these products to neuroinflammatory and vascular processes in dementia.^36^, ^37^

Emerging evidence indicates that gut microbiota, particularly opportunistic Proteobacteria, can influence oxylipin profiles by modulating host lipid‐metabolizing enzymes and by directly transforming PUFAs into LOX‐mediated oxylipins within the gut lumen. Such microbial contributions could help explain the oxylipin shifts observed here and suggest another pathway through which dysbiosis may influence inflammatory tone.^37^

We also detected changes in fecal endocannabinoids (e.g., 2‐AGs, 2‐OG, 2‐LG), pointing to alterations in the gut endocannabinoid (eCB) system, which is involved in barrier integrity, immune modulation, and gut‐brain signaling.^39^, ^40^ Given the bidirectional interactions between microbiota and eCB pathways, these findings may represent an additional link between microbial composition and host lipid signaling, but further work is needed to define their functional significance.

Correlation analyses showed that reductions in SCFA‐producing bacteria and increases in opportunistic taxa were associated with higher cytokine levels and with metabolic changes including oxylipins, long‐chain fatty acids, and glucose. This pattern is consistent with a feedback loop in which dysbiosis reduces protective SCFAs, compromises barrier function, increases luminal substrates for pro‐inflammatory lipid mediator production, and promotes peripheral inflammation with potential downstream effects on the brain.

In conclusion, several large‐scale CI metabolomic and microbiome studies have reported consistent patterns, including altered bile acid, TMAO, tryptophan metabolism, oxylipin dysregulation, reduced SCFA producers, and Proteobacteria expansion. The present findings align with this broader literature, suggesting that fecal lipid mediator and bile acid changes can be detected as early as the SCD stage, indicating that metabolic alterations begin at this stage. This highlights the importance of paying attention to SCD as a potential window for prognosis and preventive interventions. Moreover, the observed correlations between altered microbial composition, inflammatory responses, and systemic metabolic changes underscore the potential for therapeutic strategies targeting the gut‐brain axis. Confirmation in larger longitudinal cohorts will be essential to establish timing, causality, and clinical relevance. Future studies integrating metadata and dietary information (exposome) will be critical for fully understanding the interactions influencing dementia progression and for identifying pathways that could inform interventions to manage this complex neurological disorder.

### 5.1 Limitation and further work

While this study is among the few that examine different stages of cognitive impairment, from SCD to dementia, and investigate both fecal and plasma metabolites in relation to gut microbiota alterations, it also has limitations. This is a small observational study and should be considered with caution. Its limited sample size may have impacted the generalizability of the findings, and the small number of participants involved in the study limits the statistical power and may introduce potential biases or confounding factors. Moreover, some of the study information was not available for all study participants. While acknowledging its limitations, this study implies that the interactions between the microbiota, the gut, and the brain are considerably more intricate than previously presumed. To corroborate and build upon our results, it is imperative that further research be conducted with larger and more diverse study populations, accompanied by more comprehensive metadata information. Moreover, it may be valuable to stratify the SCD group in future studies into those who will develop cognitive impairment and those who won't. This could help identify specific microbial or metabolic markers that differentiate these subgroups and provide early insights into cognitive decline progression.

## CONFLICT OF INTEREST STATEMENT

The authors declare no conflicts of interest.

## CONSENT STATEMENT

Written informed consent was obtained from all participants, and the study received approval from the Geneva Ethics Committee (CCER_2016‐01346 and CCER_2020_00403)

## Supporting information



The following are available online, Table S1: Plasma Immunoassays, Table S2: Metabolomics Data analysis and Missing values, Table S3: Non‐Parametric analysis of plasma proinflammatory cytokine levels across cognitive impairment states versus healthy controls, Table S4: The total abundance of various bacterial taxa in fecal samples from individuals with different cognitive impairment statuses, Table S5: Conducting linear model estimates analysis on plasma metabolites in various cognitive impairment statuses compared to a healthy control group, Table S6: Conducting linear model estimates analysis on feces metabolites in various cognitive impairment statuses compared to a healthy control group, Table S7: Spearman correlation analyses investigating associations between alterations in plasma metabolites, along with plasma cytokine levels, and the abundance of bacteria, Table S8: Spearman correlation analyses investigating associations between alterations in fecal metabolites and the abundance of bacteria.

Figure S1: Lower and upper limits of detection for four cytokine standards measured using the Meso Scale Discovery (MSD) platform, Figure S2: Linear regression analysis of inflammatory cytokines with age and MMSE score in different statues of cognitive impairment, Figure S3: Microbiome compositional analysis of individuals in different status of cognitive impairment (based on Phylum abundance), Figure S4: Spearman correlation between the levels of metabolites in feces and their corresponding levels in plasma.

Supporting Information
