# Supplementary material for: Plasma and feces multiomics unveil cognition‐associated perturbations of chronic inflammatory pathways of the gut‐microbiota–brain axis
Source: Alzheimers Dement. 2025 Oct 30;21(10):e70844. doi: 10.1002/alz.70844 (PMC12572818; doi:10.1002/alz.70844)
Supplement: Supplementary file 2 — Figure S1: Lower and upper limits of detection for four cytokine standards measured using the Meso Scale Discovery (MSD) platform, Figure S2: Linear regression analysis of inflammatory cytokines with age and MMSE score in different statues of cognitive impairment, Figure S3: Microbiome compositional analysis of individuals in different status of cognitive impairment (based on Phylum abundance), Figure S4: Spearman correlation between the levels of metabolites in feces and their corresponding levels in plasma. [file ALZ-21-e70844-s002.pdf]

Supplementary File

Plasma extraction for profiling of polar metabolites:

1. Switch on the SpeedVac and cool centrifuge to 4°C.
2. Thaw all samples (aliquoted 100 µl to 2 mL Eppendorf tube) on ice.
3. Add 10 µL of internal standard mix to all samples except the blank. Add 10 µL of MilliQ-water to the blank samples.
4. Add 75% ice cold methanol-water (400 µL methanol and 120 µl HPLC water) per plasma sample using Eppendorf Multipipett.
5. Close all Eppendorf tube securely, vortex for 5min at speed 7.
6. Incubate samples at -20°C for 20 min
7. Transfer the samples to the centrifuge and spin down for 10 min at 4°C at 18000 RCF.
8. Afterwards, transfer the supernatant into a new Eppendorf tube and add 520 µL chloroform and 350 µL milliQ-water respectively. This step enables phase separation.
9. Let samples rest on ice for 10 min; then transfer them to the centrifuge and spin down for 10 min at 4°C at 20,000 RCF.
10. Collect the upper aqueous phase and transfer it to a clean 1.5 mL Eppendorf tube. Make sure the extracted volume is the same for each sample.
11. Dry the collection tubes in the SpeedVac at room temperature.
12. Collect the samples and add 100 µL of ice-cold injection solution (6:4 v/v MeOH: milliQ-water) to all samples and close tubes securely.
13. Shake the samples on the MultiTube Vortexer at speed 7 for 5 min.
14. Transfer the samples to the centrifuge and spin down for 10 min at 4°C at 20,000 RCF.
15. Transfer 90 µL of the sample volume into autosampler glass vials for analysis.

Faces extraction for profiling of polar metabolites

1. Switch on the SpeedVac and cool centrifuge to 4°C.
2. Thaw all samples (aliquoted 50mg of freeze-dried fecal sample to 2 mL Eppendorf tube) on ice.
3. Add 20 µL of internal standard mix to all samples except the blank. Add 10 µL of MilliQ-water to the blank samples.
4. Add 75% ice cold methanol-water (200 µL methanol and 60 µl HPLC water) per fecal sample using Eppendorf Multipipett except the calibration samples.
5. Close all Eppendorf tube securely, vortex for 5min at speed 7.
6. Incubate samples at -20°C for 20 min (extremely important when working with feces)
7. Transfer the samples to the centrifuge and spin down for 10 min at 4°C at 18000 RCF.
8. Afterwards, transfer the supernatant into a new Eppendorf tube and add 260 µL chloroform and 175 µL milliQ-water respectively. This step enables phase separation.
9. Let samples rest on ice for 10 min; then transfer them to the centrifuge and spin down for 10 min at 4°C at 20,000 RCF.

10. Collect 150 µL of the upper aqueous phase and transfer it to a clean 1.5 mL Eppendorf tube. Make sure the extracted volume is the same for each sample.
11. Dry the collection tubes in the SpeedVac at room temperature.
12. Collect the samples and add 150 µL of ice-cold injection solution (6:4 v/v MeOH: milliQ-water) to all samples and close tubes securely. For QC pool samples add 50uL.
13. Shake the samples on the MultiTube Vortexer at speed 8 for 5 min.
14. Transfer the samples to the centrifuge and spin down for 10 min at 4°C at 20,000 RCF.
15. Transfer 50 µL of the sample volume into autosampler glass vials for analysis. Positive and Negative ESI separately.

Faces extraction for profiling of signaling lipids

1. Weight 30mg of freeze-dried fine powder feces in 2mL Eppendorf tube
2. Add 5 µL of antioxidant solution with a multipette.
3. Add 20 µL of internal standard solution with a multipette.
4. Add 150 µL of citric acid/phosphate buffer with a multipette.
5. Slowly add 1 mL of the BuOH:ethylacetate (1:1 v:v) extraction solution with a multipette.
6. Close all Eppendorf tubes securely, vortex shortly and let the samples settle during 20 minutes. This will facilitate the LLE because the protein layer will become more compact.
7. Transfer the samples to the bullet blender to mix for 2 minutes at the highest speed (level 10).
8. Afterwards, transfer the samples to the centrifuge and spin down for 10 min at 4°C at 18000 RCF.
9. Using an air displacement pipette, collect 500 µL of the upper organic phase and transfer it to a clean 1.5 mL tube, the collection tube. Make sure the extracted volume is the same for each sample.
10. Dry the collection tubes overnight in the SpeedVac at ambient temperature.
11. Collect the samples on the next day and add 50 µL of ice-cold injection solution with a multipette.
12. Close the tubes quickly and securely after reconstitution.
13. Shake the samples on the MultiTube Vortexer at medium speed (7) for 2 minutes followed at high
14. speed (10) for 2 minutes.
15. Transfer the samples to the centrifuge and spin down for 10 min at 4°C at 15700 RCF.
16. Using an air displacement pipette, transfer 50 µL of the sample volume into autosampler glass vials for analyses.

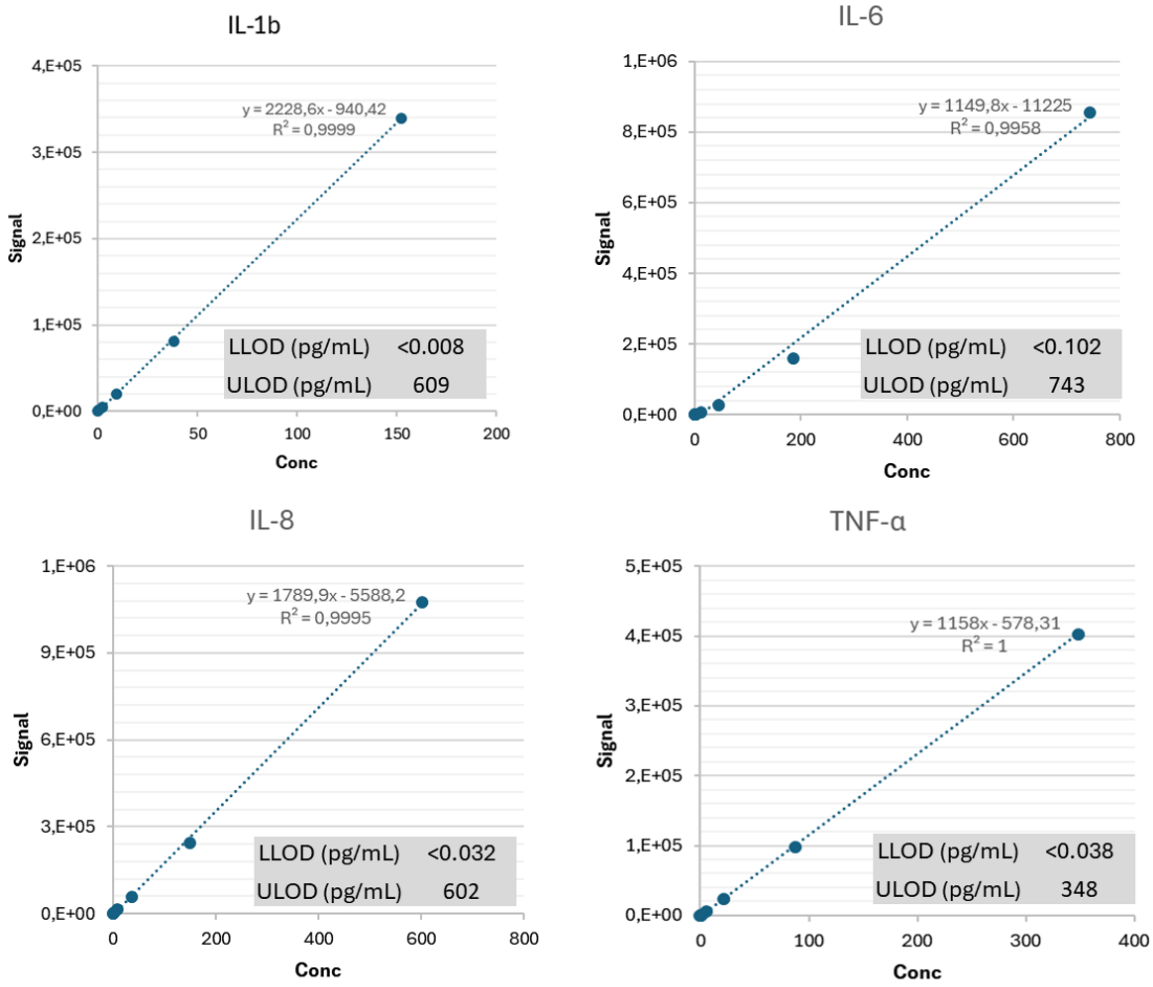

***Supplementary Figure S1*. Lower and upper limits of detection for four cytokine standards measured using the Meso Scale Discovery (MSD) platform.**

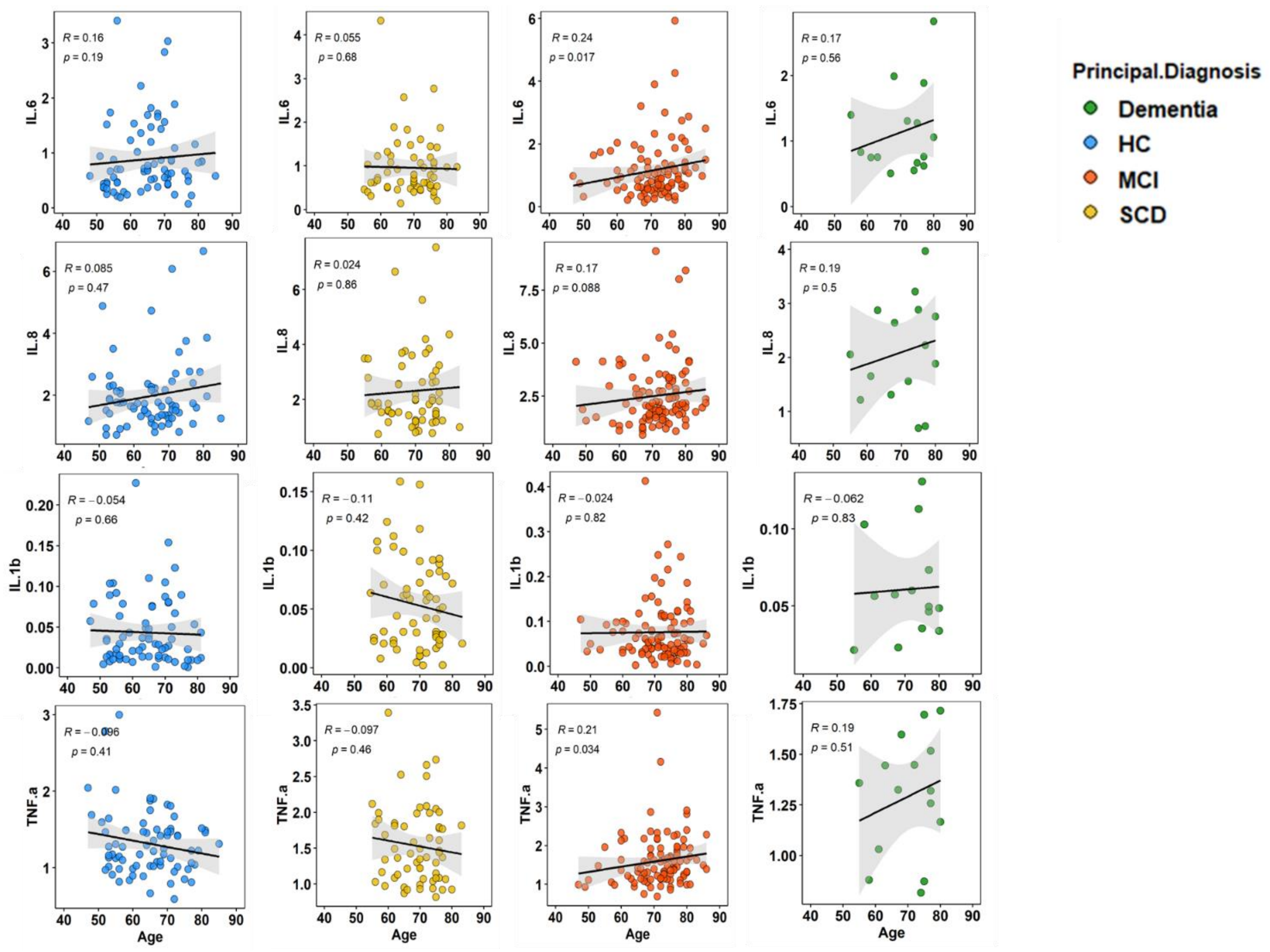
R = 0.16
p = 0.19
R = 0.055
p = 0.68
R = 0.24
p = 0.017
R = 0.17
p = 0.56
R = 0.085
p = 0.47
R = 0.024
p = 0.86
R = 0.17
p = 0.088
R = 0.19
p = 0.5
R = −0.054
p = 0.66
R = −0.11
p = 0.42
R = −0.024
p = 0.82
R = −0.062
p = 0.83
R = −0.096
p = 0.41
R = −0.097
p = 0.46
R = 0.21
p = 0.034
R = 0.19
p = 0.51
IL.6
IL.8
IL.1b
TNF.a
Age
Principal.Diagnosis
Dementia
HC
MCI
SCD

a.

b.

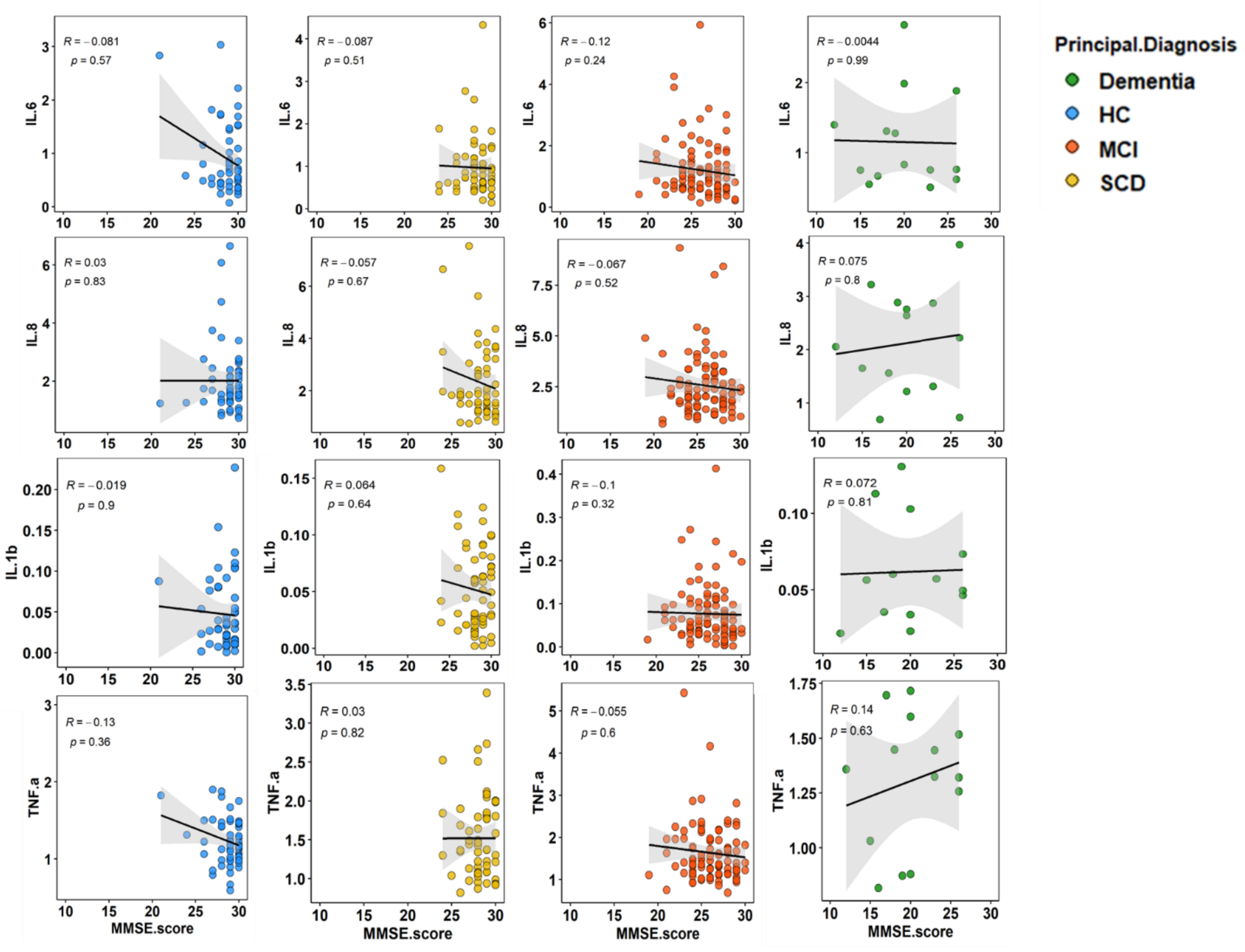

Supplementary Figure S2*. Linear regression analysis of plasma inflammatory cytokines with age (A) and MMSE score (B) in different statues of cognitive impairment*

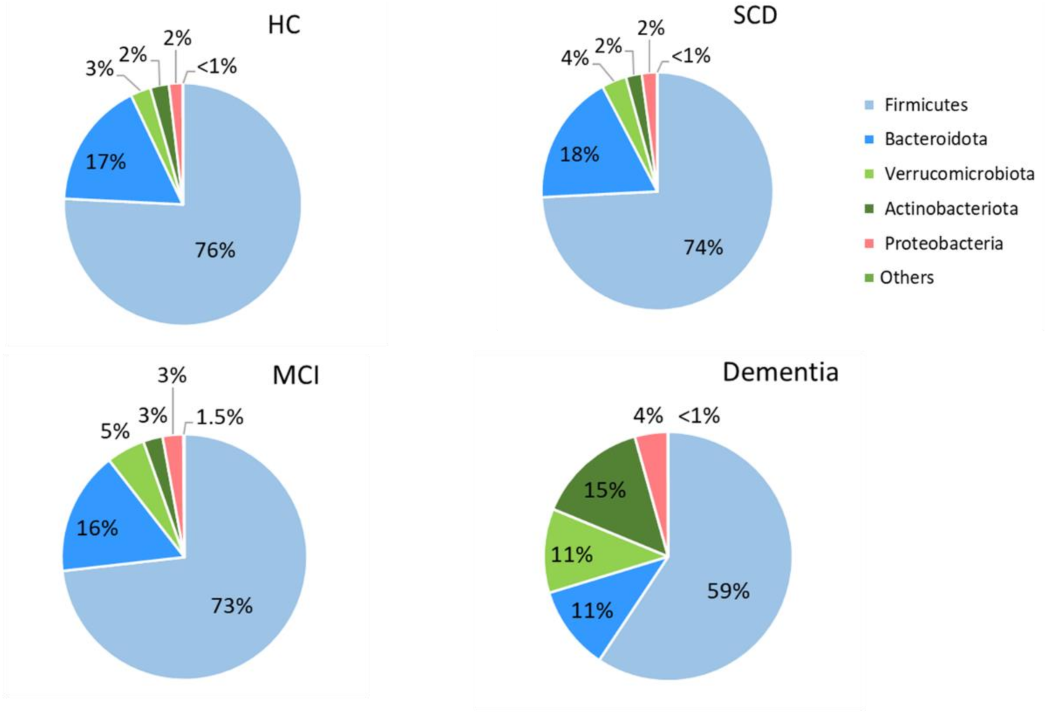

***Supplementary Figure S3**. Microbiome compositional analysis of individuals in different status of cognitive impairment (based on Phylum abundance)*

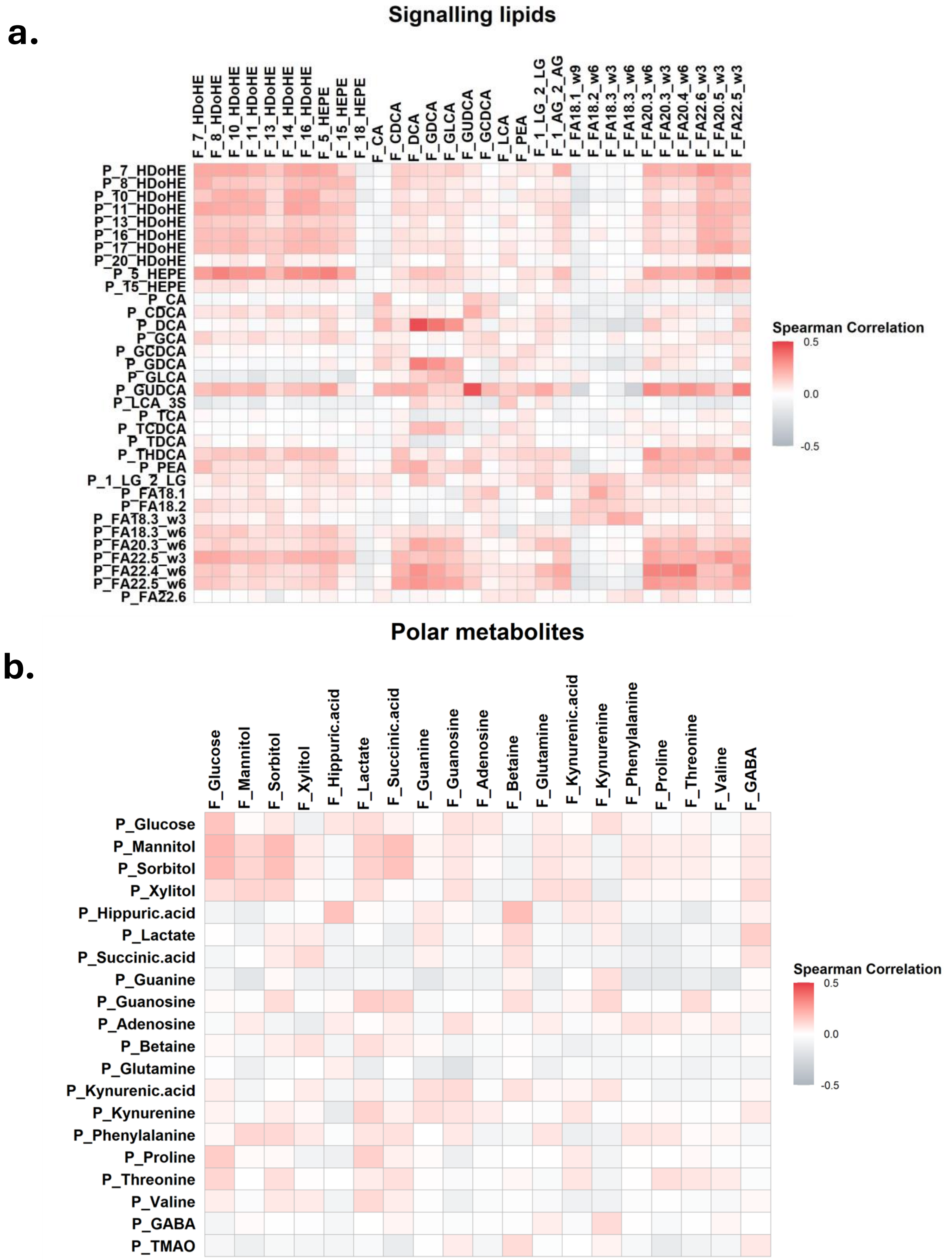

Supplementary Figure S4. *Spearman correlation between the levels of metabolites in feces and their corresponding levels in plasma. a. Signaling lipids, b. Polar metabolites.* ***a.*** *Signaling lipids,* ***b.*** *Polar metabolites. P: stands for metabolites in plasma, F: stands for metabolites in feces*
